# Influence of the Polymer Microstructure over the Phase Separation of Thermo-Responsive Nanoparticles

**DOI:** 10.3390/polym13071032

**Published:** 2021-03-26

**Authors:** Nicolò Manfredini, Marco Tomasoni, Mattia Sponchioni, Davide Moscatelli

**Affiliations:** Department of Chemistry, Materials and Chemical Engineering “Giulio Natta”, Politecnico di Milano, via Mancinelli 7, 20131 Milan, Italy; nicolo.manfredini@polimi.it (N.M.); marco3.tomasoni@mail.polimi.it (M.T.); davide.moscatelli@polimi.it (D.M.)

**Keywords:** polymeric nanoparticles, emulsion polymerization, RAFT, thermo-responsive polymers, smart materials, LCST, phase diagram, phase separation

## Abstract

Thermo-responsive nanoparticles (NPs), i.e., colloids with a sharp and often reversible phase separation in response to thermal stimuli, are coming to the forefront due to their dynamic behavior, useful in applications ranging from biomedicine to advanced separations and smart optics. What is guiding the macroscopic behavior of these systems above their critical temperature is mainly the microstructure of the polymer chains of which these NPs are comprised. Therefore, a comprehensive understanding of the influence of the polymer properties over the thermal response is highly required to reproducibly target a specific behavior. In this study, we synthesized thermo-responsive NPs with different size, polymeric microstructure and hydrophilic-lipophilic balance (HLB) and investigated the role of these properties over their phase separation. We first synthesized four different thermo-responsive oligomers via Reversible Addition-Fragmentation Chain Transfer (RAFT) Polymerization of poly(ethylene glycol)methyl ether methacrylate. Then, exploiting the RAFT living character, we chain-extended these oligomers with butyl methacrylate obtaining a library of NPs. Finally, we investigated the NP thermo-responsive behavior, their physical state above the cloud point (Tcp) as well as their reversibility once the stimulus is removed. We concluded that the solid content plays a minor role compared to the relative length of the two blocks forming the polymer chains. In particular, the longer the stabilizer, the more favored the formation of a gel. At the same time, the reversibility is mainly achieved at high HLB, independently from the absolute lengths of the block copolymers.

## 1. Introduction

Polymeric nanoparticles (NPs) have been extensively studied since the middle of the previous century for their appealing and controllable interfacial and composition properties [1,2,3]. These features determined their consolidation in several fields, such as coating [4], painting [5], textile [6], or cosmetics [7].

Nowadays, these colloids are mainly produced via free radical emulsion polymerization (FRPe) [8]. This technique allows the synthesis of concentrated (up to 50–60% *w/w*) NP suspensions, commonly referred to as latexes, directly in water, avoiding the use of any organic solvent [9,10]. Although economically viable and brought to a multi-ton scale to satisfy the ever-increasing market demand for polymer latexes, one of the main drawbacks of FRPe is the limited control over the polymer microstructure and hence over the NP physico-chemical properties [11,12].

In the last decades, this limitation was progressively overcome by the advent of controlled radical polymerizations, which gave new lymph to the field [13,14,15]. In particular, the high control over the polymer chain microstructure achievable with these polymerization techniques paved the way to highly engineered colloids [16,17,18,19,20,21,22], contributing to the spreading of the NP technology towards previously unexplored applications.

In a similar direction, such controlled radical polymerizations were exploited to tune the response of a particularly appealing class of NPs, the so-called stimuli-responsive NPs, i.e., colloids with a sharp and often reversible phase separation behavior in response to external stimuli. Among the different stimuli that can be exploited to trigger such behavior (e.g., pH [23], temperature [24,25,26,27,28], CO_2_ [29,30], redox potential [31]), the temperature is surely one of the most interesting, promising and versatile. In fact, the possibility of applying thermal stimuli in a controlled way, coupled with the natural occurrence of thermal gradients in living organisms, made thermo-responsive materials attractive for several applications.

In the literature, two opposite behaviors are reported for thermo-responsive polymers. With reference to the phase diagram, namely the diagram of temperature vs. polymer volume fraction, for a solvent/polymer binary mixture, the thermo-responsive polymers can be miscible with the solvent in any proportion below their binodal curve, while phase separating in a polymer-rich phase above it. The minimum of this curve is called lower critical solution temperature (LCST), while each other point is commonly referred to as cloud point (Tcp), since the formation of polymer-rich droplets above this critical temperature often determines the cloudiness of the system. Oppositely, the binodal curve can form a maximum, known as upper critical solution temperature (UCST), with the thermo-responsive polymer being miscible with the solvent above this curve, while phase separating below it [24,32,33]. Despite examples of both kinds of systems have been reported, the easier capability of modulating the Tcp and the almost insensitivity of their phase behavior to environmental properties rendered polymers with a LCST the most studied. These have been successfully investigated in many different applications ranging from pharmaceutical [17,24,34,35,36] to oil and gas [37] industries.

What clearly appears from these studies is that the peculiar microstructure of the chains constituting the final NPs has a tremendous impact on the macroscopic thermo-responsive behavior of these colloids, including their reversibility (i.e., the capacity to recover the original physico-chemical properties when the stimulus is removed) [38]. At the same time, guidelines for the selection of the polymer chain requisites necessary to access a desired macroscopic behavior are lacking.

**Scheme 1:**
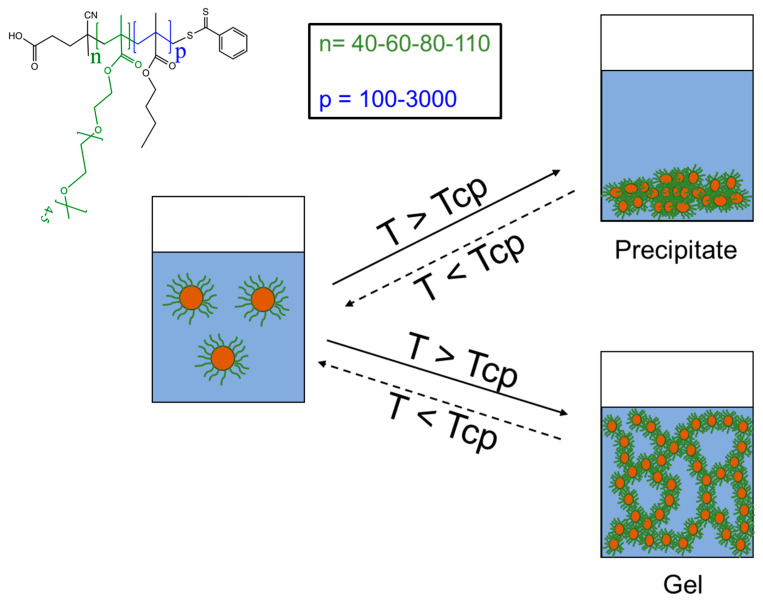
Schematic representation of the expected thermo-responsive behavior for NPs stabilized by a thermo-responsive polymer.

In this work, with the aim of rationalizing this concept, we considered NPs constituted by amphiphilic block copolymers comprising a thermo-responsive chain and a hydrophobic portion and systematically investigated the impact of the length of each chain on the final thermo-responsive behavior. In particular, we first synthesized four different thermo-responsive macromolecular chain transfer agents (macro CTAs) via reversible addition-fragmentation chain transfer (RAFT) polymerization. This technique allows to set a priori the average degree of polymerization (*n*) of the oligomers by simply changing the thermo-responsive monomer (in our case, oligo(ethylene glycol)_4_ methyl ether methacrylate, hereinafter EG_4_) to chain transfer agent (CTA) molar ratio. The choice of this thermo-responsive monomer instead of the more common N-isopropylacrylamide (NIPAM) was driven by the higher biocompatibility and poor hysteresis of EG_4_ that contributed to intensify the research towards this compound [17]. Then, exploiting the living nature of the RAFT polymerization, we chain-extended these macro CTAs with butyl methacrylate (BMA) and targeted different chain lengths (*p*). The adoption of the RAFT emulsion polymerization allowed us to obtain the formation of NPs with a lipophilic core and a thermo-responsive shell (nEG_4_-pBMA) directly in water. These colloids are expected to be stable as long as the temperature is kept below the Tcp. In fact, in this configuration the thermo-responsive chains should be well hydrated providing steric stabilization to the colloids. On the contrary, as soon as the temperature is raised above the Tcp, a sharp coil-to-globule transition of the EG_4_ moieties is expected. With the thermo-responsive chains collapsing on the NP surface, the colloidal stability is lost, which leads to the NP aggregation, as schematically reported in Scheme 1. For each combination of *n* and *p*, we evaluated the physical state (precipitate or gel in Scheme 1) of the separated phase as well as the reversibility of these aggregates once the temperature is lowered again below the Tcp and concluded on the factors governing such macroscopic behavior.

## 2. Materials and Methods

### 2.1. Materials

4-Cyano-4-(phenyl-carbonothioylthio) pentanoic acid (CPA, MW = 279.38, 99%, Sigma Aldrich, Saint Louis, MO, USA), 2,2′-azobis(2-methylpropionamidine)dihydrochloride (V-50, MW = 271.19, 98%, Acros Organics, Geel, Belgium), poly(ethylene glycol)methylether methacrylate (EG_4_, MW = ca300, Sigma Aldrich, Saint Louis, MO, USA), 4-4′-azobis (4-cyanovaleric acid) (ACVA, MW = 280.28, Sigma Aldrich, Saint Louis, MO, USA), butyl methacrylate (BMA, ≥99%, MW = 142.20, Fluka Chemika, Buchs, Switzerland), diethyl ether (99.7%, MW = 74.12, Sigma Aldrich, Saint Louis, MO, USA), deuterated chloroform (CDCl_3_, 99.8%, MW = 120.38, Sigma Aldrich, Saint Louis, MO, USA), ethanol (EtOH, MW = 46.07, ≥99.8%, Sigma Aldrich, Saint Louis, MO, USA), and tetrahydrofuran (THF, ≥99.9%, MW = 72.11, Sigma Aldrich, Saint Louis, MO, USA) were used as received.

### 2.2. Synthesis of the Thermo-Responsive Macro CTAs

Four different thermo-responsive macro CTAs were synthesized via RAFT polymerization of EG_4_ using ACVA as initiator and CPA as CTA. The ACVA to CPA molar ratio (IA) was kept equal to 1/3 for all the syntheses while the EG_4_ to CPA molar ratio (*n*) was varied in order to obtain different degrees of polymerization (i.e., *n* = 40, 60, 80, and 100). The polymerizations were carried out in ethanol at a monomer concentration equal to 20% *w/w*. Briefly, to synthesize the macro CTA with *n* = 80, hereinafter 80EG_4_, 8 g of EG_4_ (26.6 mmol), 0.093 g of CPA (0.33 mmol, i.e., EG_4_/CPA = 80 mol/mol), and 0.031 g of ACVA (0.11 mmol, i.e., IA = 1/3 mol/mol) were placed in a round bottom flask equipped with a magnetic stirrer. Then, 32.5 g of ethanol were added and the solution was purged with nitrogen for 20 min. The reaction was let occurring at 65 °C for 24 h under stirring at 300 rpm. The solution was then purified through three consecutive precipitations in diethyl ether in order to remove all the impurities and the unreacted monomer. At the end of the purification process, the solution was centrifuged for 10 min at 5000 rpm to separate the two phases and the polymer pellet was recovered and dried inside a vacuum drying oven (VACUCELL) at 35 °C overnight. Finally, the polymer was stored at −20 °C. Aliquots of the samples were dissolved in deuterated chloroform (CDCl_3_) at a concentration equal to 14 mg/mL and analyzed via nuclear magnetic resonance (^1^H-NMR) on a Bruker Ultrashield 400 MHz spectrometer (Bruker, Billerica, MA, USA) with 64 scans (see reaction scheme and spectrum in Figure S1). The monomer conversion and average degree of polymerization, *n*, were calculated according to Equations (S1) and (S2), respectively, and the results are summarized in Table S1.

In order to calculate the number-average molecular weight (*Mn*) and dispersity (*Đ*), the samples were dissolved in tetrahydrofuran (THF) at a concentration of 4 mg/mL, filtered through a 0.45 μm polytetrafluoroethylene (PTFE) membrane and injected in a Jasco LC-2000Plus (Jasco, Easton, MD, USA) gel permeation chromatograph (GPC). The analysis was performed at a flow rate of 1 mL/min and at a temperature equal to 35 °C. Three styrene/divinyl benzene columns in series (300 × 8 mm^2^, particle size 5 μm, pore size 1000, 10^5^ and 10^6^ A, respectively) were used for the separation and the signal collected with a refractive index (RI) detector. A pre-column (50 × 8 mm^2^) was added before the first column. The instrument calibration was performed with polystyrene standards.

### 2.3. Synthesis of Amphiphilic Block Copolymers Self-Assembled into NPs

The thermo-responsive macro CTAs were chain-extended with butyl methacrylate via RAFT emulsion polymerization in order to produce amphiphilic block copolymers self-assembled into NPs. These block copolymers, hereinafter nEG_4_-pBMA, were synthesized setting the initiator to macro CTA molar ratio to 1/3 and varying the BMA to macro CTA molar ratio (*p*) in order to obtain copolymers with different degrees of polymerization. The polymerization was performed in water at a concentration equal to 20% *w/w*.

For example, in the synthesis of 80EG_4_-500BMA (i.e., molar ratio between BMA and 80EG_4_ equal to 500), 1 g of BMA (7 mmol), 0.363 g of 80EG_4_ (0.015 mmol, i.e., BMA/80EG_4_ = 500 mol/mol), and 1.27 mg of V-50 (0.005 mmol, i.e., V-50/80EG_4_ = 1/3 mol/mol) were mixed in 3.64 g of distilled water. The suspension was purged with nitrogen for 20 min at room temperature to avoid side reactions, mixed using a vortex and placed in a pre-heated oil bath at 50 °C under magnetic stirring for 24 h.

At the end of the reaction, the NP suspensions were placed in a cellulose membrane (Spectra/Por) with a molecular weight cut-off of 3.5 kDa and were dialyzed against distilled water for 48 h.

The reaction conversion was first evaluated via thermogravimetric analysis performed on an Ohaus MB35 moisture analyzer [6] and then confirmed via ^1^H-NMR (Figure S2, Equation (S3)) following the same procedure described in Section 2.2. From the ^1^H-NMR spectrum, the actual *p* was calculated according to Equation (S4). The block copolymer *Mn* and *Đ* (Table S2) were measured via GPC according to the procedure described in Section 2.2.

The NP size (reported in terms of Z-average, indicated as Dn) and polydispersity index (PDI) were measured via dynamic light scattering (DLS) on a Zetasizer Nano ZS (Malvern, UK) at a scattering angle of 173° (Table 1). Each sample was diluted before the analysis to a concentration equal to 1% *w/w* and analyzed in triplicate with 13 runs per measurement.

The block copolymer hydrophilic-lipophilic balance (HLB, Table 1) was calculated according to Equation (1):

(1) $$HLB=20\frac{M_{h}}{M_{t}}$$

where $M_{h}$ is the molecular mass of the hydrophilic portion of the molecule (i.e., the thermo-responsive portion in our case), and $M_{t}$ is the molecular mass of the whole molecule. This value is a number comprised within 0–20 with a value of 0 corresponding to a completely lipophilic molecule and a value of 20 corresponding to a completely hydrophilic molecule.

### 2.4. Study of the Thermo-Responsive Properties

The NP cloud point (Tcp) was calculated via DLS analyzing the inflection points of Dn vs. temperature curves [17]. The temperature was ramped from 25 °C up to 70 °C at intervals of 1 °C, leaving 120 s of equilibration time before each measurement.

In order to evaluate the NP reversibility, all the samples were diluted at 20%, 10%, 5%, and 1% *w/w* and placed in a pre-heated oven at 80 °C for 1 h. At the end of the heating procedure, the NPs were removed and left to equilibrate at room temperature for an additional hour.

The samples showing a reversibility (i.e., turned again cloudy) were analyzed via DLS in order to compare their actual size with the original one (i.e., before the heating procedure).

## 3. Results and Discussion

Thermo-responsive amphiphilic block copolymers with a well-defined microstructure and self-assembled into NPs were synthesized via two consecutive RAFT polymerizations.

**Figure 1:**
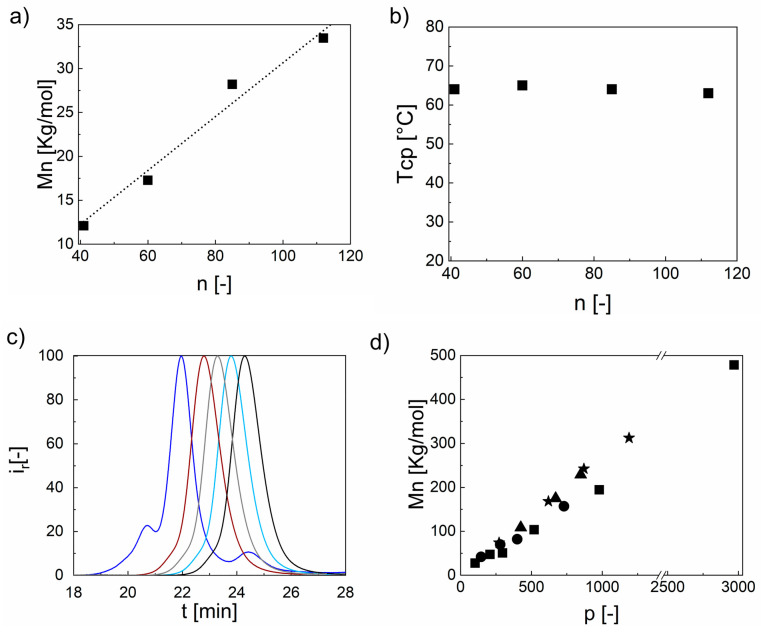
(**a**) Number average molecular weight as function of the degree of polymerization of the thermo-responsive block (**b**) Tcp as a function of the degree of polymerization of the thermo-responsive block (**c**) Normalized chromatograms of the block copolymers synthesized from 100EG_4_ having p equal to 0 (black), 275 (light blue), 600 (grey), 850 (red), and 1200 (blue), respectively. i_r_ represents the normalized intensity of the refractive index detector. (**d**) Number-average molecular weight as a function of the degree of polymerization of the lipophilic block for polymers having *n* equal to 40 (square), 60 (circle), 80 (triangle), and 100 (star), respectively.

We first synthesized four thermo-responsive macro CTAs (nEG_4_, with *n* equal to 40, 60, 80, and 100, respectively) through RAFT solution polymerization of EG_4_ in ethanol. High monomer conversion (>94%), poor *Đ* (<1.2) and controllable *Mn* were obtained (see Table S1 in the Supporting Information). In fact, one peculiarity of the RAFT process is the possibility of setting *a priori* the desired molecular weight by simply changing the monomer to CTA molar ratio (*n*) as shown in Equation (2) [39].

(2) $$M_{n_{m\mathrm{CTA}}}=M_{\mathrm{CTA}}+nXM_{EG_{4}}$$

being *X* the monomer conversion, $M_{n_{m\mathrm{CTA}}}$, *M*_CPA_ and *M*_EG_4__ the molecular weights of the produced macro CTA (i.e., number-average molecular weight), CTA and EG_4_ respectively. This Equation is validated by the linear increase in *M_n_* reported in Figure 1a for different values of *n*. At the same time, the RAFT polymerization ensures that the actual *n* of the product, as measured through ^1^H-NMR, closely matches the target value, as shown in the comparative plot reported in Figure S3a, thus granting a good control over the polymer average chain length.

It is worth noticing that this increase in the polymer molecular weight is not followed by a change in its Tcp, which changed in a very narrow range between 62 and 66 °C, as demonstrated in Figure 1b. The phase separation at this temperature may find application in the oil and gas filed, where thermo-responsive polymers can be applied as viscosity modifiers or to enhance the emulsification/separation of oil in water during the so-called enhanced oil recovery [37,40,41]. However, we would like to point out that with this work we are not aiming at a particular application. Rather, EG_4_ was selected as a representative of the oligo(ethylene glycol)methyl ether methacrylates, with a controllable phase separation. The Tcp of these polymers can be readily adjusted to the desired value by choosing the appropriate oligo(ethylene glycol) chain length or by copolymerization with hydrophilic (i.e., Tcp increases) or hydrophobic (i.e., Tcp decreases) monomers [24]. The poor sensitivity of the Tcp to the molecular weight for this class of polymers, quite expected for LCST-type polymers at least for these low values of *n*, allows to obtain a portfolio of macro CTAs with comparable Tcp and different *Mn*.

These macro CTAs were then chain-extended with BMA via RAFT emulsion polymerization to obtain amphiphilic block copolymers self-assembled into NPs. Different degrees of polymerization for the polyBMA portion (*p*) were targeted. Again, the RAFT polymerization ensured to achieve values of *p* very close to the target (Figure S3b). In addition, all the copolymers synthesized presented high monomer conversion (>95%) and poor *Đ* (<1.4, Table S2) as expected from a controlled radical polymerization. The possibility of forming well-defined block copolymers is further demonstrated by the high blocking efficiency. In fact, in most of the samples, the macro CTA showed high conversion during the polymerization as its characteristic peak is strongly reduced in intensity in the GPC chromatogram of the block copolymers. This is shown as an example in Figure 1c for the 100EG_4_-*p*BMA samples. The peak of the macro CTA at 24.5 min is seen as a shoulder in the chromatogram of the 100EG_4_-1200BMA, while it is not separated and resolved in all the other samples, suggesting high conversion. Indeed, for these samples, low molecular weight tails that could hide the signal from the macro CTA were recorded. However, their intensity is quite small compared to that of the main copolymer distribution, suggesting that in the worst scenario the residual macro CTA concentration is low as well.

This two-step RAFT polymerization strategy provides two degrees of freedom (*n* and *p*) enabling to independently control the partition of the thermo-responsive and hydrophobic units and the copolymer *Mn,* according to Equation (3) (Figure 1d):

(3) $$M_{n}=M_{n_{m\mathrm{CTA}}}+pXM_{\mathrm{BMA}}=M_{\mathrm{CTA}}+nM_{EG_{4}}+\;pXM_{\mathrm{BMA}}$$

with $M_{\mathrm{BMA}}$ and $M_{n}$ being the BMA and final block-copolymer number-average molecular weight, respectively. In particular, through the tuning of *n* and *p* it is possible to produce copolymers with the same *Mn* and different hydrophilic-to-lipophilic balance (HLB). To rationalize this concept, we calculated for all the samples (Table 1) the corresponding HLB (Equation (1)), which was found to play a relevant role in dictating the macroscopic thermo-responsive behavior, as discussed in the following. The HLB could be varied in the range 1–10 and, therefore, block copolymers from very hydrophobic to slightly hydrophilic could be produced with this approach.

Another advantage of using the RAFT emulsion polymerization is the simultaneous synthesis of the copolymer and its self-assembly into NPs at high solid content. The high control in the block copolymer synthesis allows the formation of colloids with tunable size (Dn) and poor polydispersity indexes (PDI) as clearly shown in Table 1.

**Figure 2:**
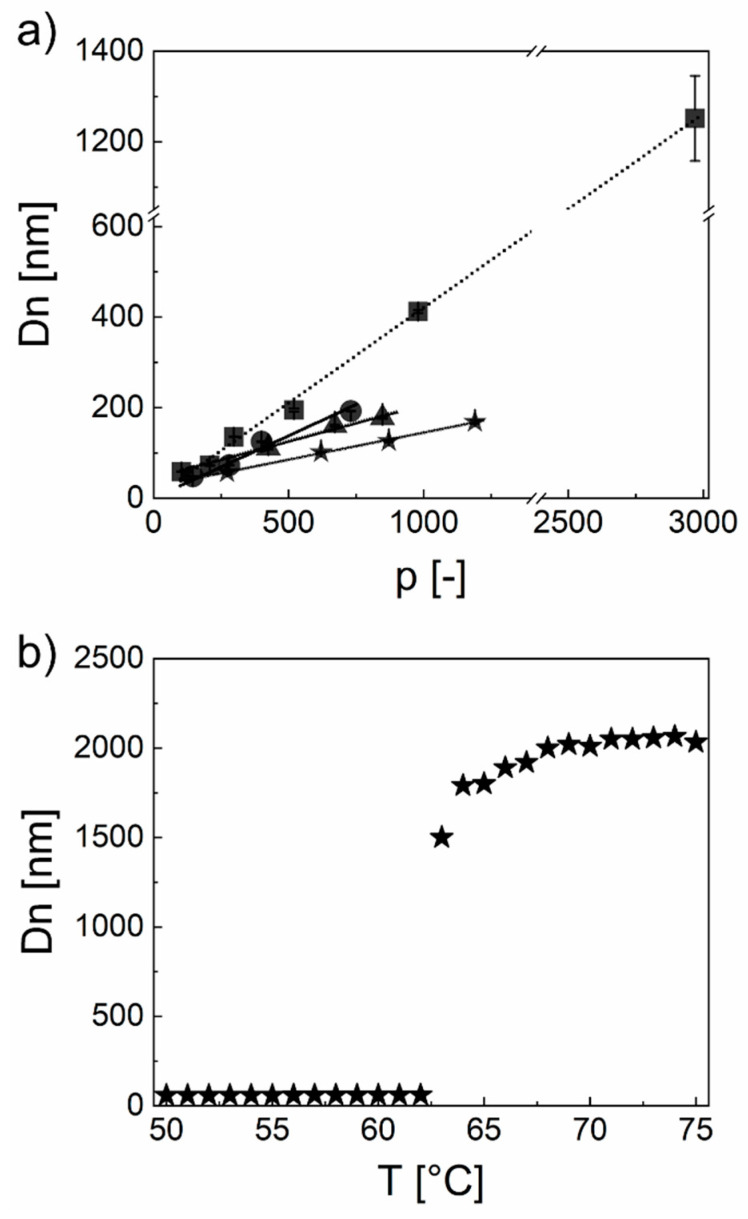
(**a**) NP size (Dn) as a function of *p* in the case of *n* equal to 40 (square), 60 (circle), 80 (triangle), and 100 (star), respectively. (**b**) NP size as function of external temperature in the case of 80EG_4_-650BMA.

In particular, the two-step approach adopted in this work makes the NP size a function of *n* and *p* (Equation (4)) [39], thus providing the opportunity of decoupling Dn from the polymer molecular weight and in turn of obtaining NPs with comparable size but different microstructure:

(4) $$D_{n}=\frac{6\;p\;M_{\mathrm{BMA}}\;}{A\;N\;\rho }=\;\frac{6\;p\;M_{\mathrm{BMA}}\;}{\left(\alpha +n\beta \right)\;N\;\rho }$$

with $M_{\mathrm{BMA}}$ being the molecular weight of BMA, ρ the density of the lipophilic chains, N the Avogadro number, A the area on the NP surface covered by a single polymeric chain, α and β two constants characteristic of the polymer. In particular, *A* is a function of the molecular weight of the hydrophilic portion of the block copolymer when amphiphilic stabilizers as *n*EG_4_ are used at a concentration higher than their critical micellar concentration (CMC). In this case, the lipophilic backbone is expected to dispose tangentially to the NP surface with the hydrophilic moieties exposed to the bulk. In a similar conformation, the higher *n* (and in turn *Mn* of the stabilizer), the higher the portion of the surface that it is able to cover and, in turn, the lower the average NP size [39]. The NP synthesized in this work are in line with this prediction and their size, at given *p*, decreases with *n* as clearly visible in Figure 2a. In fact, the higher *n* the higher the surface area covered by a single chain with a consequent reduction in the NP size (Equation (4)). Moreover, a linear increase in the NP size with *p*, at a given *n*, is obtained independently from the stabilizer used (Figure 2a).

It is worth mentioning that a broad range of Dn can be accessed with this strategy, from around 50 nm up to 1 μm (i.e., 40EG_4_-3000BMA). This is a remarkable result for colloids produced via RAFT emulsion polymerization, which typically leads to NPs with an upper size limit of 500 nm [42].

The high control achievable with this two-step RAFT polymerization strategy allows the formation of a wide portfolio of colloids with different size and HLB suitable for a systematic analysis of the impact of these parameters over the macroscopic thermo-responsive behavior.

First, we measured the Tcp of the different samples via DLS, according to the procedure described in Section 2.4. All the NPs show a similar trend with a sharp change in their size from nm to few µm once the Tcp is reached (Figure 2b). It is worth mentioning that the absolute value of the size reached above the Tcp should be only considered as an indication, since at this size scale the gravity causes the sedimentation of the larger aggregates already within the time of the analysis. What is reliable is the dynamic of the phase separation, which is due to the loss of steric stability. In fact, once the Tcp is overcome, the thermo-responsive shell collapses on the NP core leading to aggregation (Scheme 1). It is worth noticing that the Tcp is independent from *p* for all the *n* considered (Table 1). In fact, the high conversions obtained in the synthesis of the stabilizer coupled with the high compartmentalization achievable with the RAFT emulsion polymerization permits to obtain a library of NPs with a transition temperature that is dependent only on the thermo-responsive monomer chosen [17,18,19,20,21,22,23,24,25].

After having demonstrated that all of the samples exhibit a thermo-responsive behavior, their physical state above the Tcp as well as their reversibility once the thermal stimulus is removed were systematically investigated and related to the three degrees of freedom characterizing the system, i.e., solid content, *n* and *p*.

**Figure 3:**
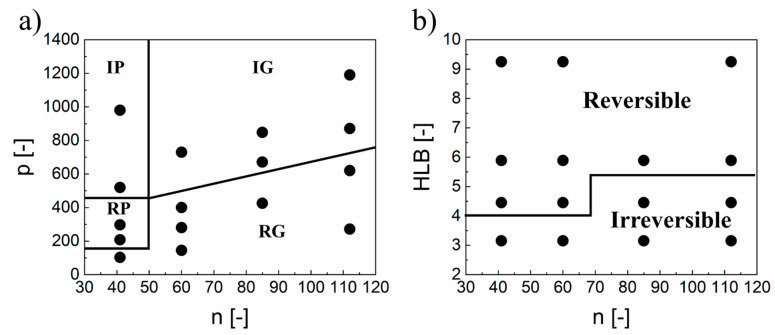
(**a**) Phase diagram reporting the physical state of the NP suspension above its Tcp at a fixed concentration of 20% *w/w* being RP, reversible precipitate, IP, irreversible precipitate, IG, irreversible gel, and RG, reversible gel, respectively. (**b**) Phase diagram reporting the reversibility of the phase separation at a given concentration of 20% *w/w* and as a function of the HLB of the block copolymer and *n*.

We explored the thermo-responsive behavior at four different concentrations, namely 1, 5, 10, and 20% *w/w* according to the procedure described in Section 2.4. Interestingly, no significant effects of NP concentration over their reversibility were noticed for all of the NPs analyzed, as visible in Figure S4. Thus, samples with a reversible transition at given *n* and *p*, are so at any of the concentrations tested. Moreover, only a modest effect on the NP physical state above the Tcp was observed with a tendency to the formation of gel when the concentration is increased. Then, the effect of *n* and *p* was investigated in details at a fixed concentration of 20% *w/w*. We divided the phase diagram into four classes depending on the NP reversibility as well as the physical state of the aggregate above the Tcp. In particular, we individuated two possible physical states, namely precipitate and gel (Figure S5). In the former, the destabilization led to the NP aggregation and precipitation of a bulky polymer phase, while in the case of the gel, the destabilization involved the whole mass of the sample, leading to a self-standing monolith. Both of these states can be reversible or irreversible depending on the capability of the NPs to recover their original size once the thermal stimulus is removed, as shown for example in Figure S6 for the sample 60EG_4_-150BMA. The four different behaviors are indicated in the phase diagram of Figure 3a as reversible precipitate, irreversible precipitate, irreversible gel, and reversible gel, respectively. In general, we observed that an increase in *p* always led to a progressive loss in the reversibility of the phase separation, independently from the *n* considered. This behavior can be justified considering that an increase in *p* is associated with an increase in the NP lipophilicity, leading to stronger hydrophobic interactions between the aggregated NPs. This hypothesis is supported by the evidence that by counter-balancing the increase in lipophilicity of the block copolymer with an increase in the thermo-responsive portion (i.e., *n*), the range of *p* leading to reversible transitions is expanded. In addition, *n* seems to play a relevant role in the physical state of the phase separated system, favoring the formation of hydrogels. This may be attributed to the decrease in the NP size, and then to an increase in their number, associated with an increase in *n*, when the solid content is fixed. This higher number of particles, together with the partial shell interpenetration could lead to a percolating system, in which at a critical NP distance the system stops flowing forming a self-standing gel [43].

Since, as shown, a change in *p* and *n* is always accomplished with a variation in the NP size making the understanding of the real impact of these parameters difficult, we re-analyzed the samples as a function of their HLB (Equation (1)). Interestingly, it was found that NPs with comparable HLB present similar size (Figure S7). Moreover, the decreasing trend of Dn with the HLB observed for all the *n* considered is in line with Equation (4). Grouping NPs with similar size allows to understand the impact of the absolute length of both blocks on the NP reversibility. In fact, as visible from Figure 3b, for HLB lower than 4.5, an irreversible aggregation is obtained independently from the *n* considered. However, the systems stabilized by shorter *n* (40 and 60 in Table S1) are more prone to the reversibility (achieved at lower HLB compared to the samples with *n* = 80 and 100). This result suggests that the longer the stabilizer, the lower the NP reversibility when size and lipophilicity are fixed, which may be due to a certain interaction between the thermo-responsive shells. Hence, a fine tuning of the block copolymer microstructure allows to decouple the NP size and HLB from their thermo-responsive response.

## 4. Conclusions

In this work, we correlated the macroscopic behavior of thermo-responsive NPs with the microstructure of the block copolymer they are made up of. The use of two sequential RAFT polymerization steps allows the synthesis of colloids with high blocking efficiency and controllable properties including size, molecular weight, HLB and microstructure. Moreover, exploiting the two degrees of freedom that this procedure allows, it was possible to decouple all the NP properties in order to better clarify the role of each parameter over the final macroscopic behavior.

First, it was found that the solid content does not play a significative role on the properties of these systems. In general, a decrease in the nanoparticle size is accomplished with a better reversibility of the system and a major tendency to form gel. However, this behavior is impacted also from the absolute length of both the portions (*n* and *p*) of the block copolymers that form the NP. In fact, NPs with comparable HLB and size recover their reversibility more easily when the stabilizing block is shorter.

## Figures and Tables

**Table 1:** Size (Dn), polydispersity index (PDI) and temperature of cloud point (Tcp) of the NP synthesized.

Sample	Dn [nm]	PDI [-]	Tcp [°C]	HLB [-]
40EG_4_-100BMA	58.9 ± 0.3	0.15 ± 0.01	64	9.1
40EG_4_-200BMA	72.6 ± 0.7	0.11 ± 0.01	65	5.9
40EG_4_-300BMA	135.9 ± 0.7	0.12 ± 0.01	63	4.5
40EG_4_-500BMA	194.9 ± 3.1	0.08 ± 0.01	63	2.8
40EG_4_-1000BMA	412.5 ± 3.5	0.09 ± 0.01	64	1.6
40EG_4_-3000BMA	1251.8 ± 94.1	0.07 ± 0.01	62	0.6
60EG_4_-150BMA	48.6 ± 0.2	0.15 ± 0.03	65	9.3
60EG_4_-275BMA	73.3 ± 0.5	0.05 ± 0.02	65	6.2
60EG_4_-400BMA	125.2 ± 0.8	0.03 ± 0.02	64	4.8
60EG_4_-725BMA	192.7 ± 0.1	0.06 ± 0.02	64	2.9
80EG_4_-450BMA	110.3 ± 2.4	0.22 ± 0.01	64	5.9
80EG_4_-650BMA	160.6 ± 2.1	0.05 ± 0.01	63	4.2
80EG_4_-850BMA	177.3 ± 1.8	0.04 ± 0.08	63	3.5
100EG_4_-275BMA	57.7 ± 2.1	0.05 ± 0.10	66	9.2
100EG_4_-600BMA	102.1± 0.9	0.03 ± 0.01	64	5.9
100EG_4_-850BMA	126.6 ± 2.5	0.03 ± 0.03	65	4.5
100EG_4_-1200BMA	168.9 ± 4.1	0.03 ± 0.04	64	3.1

## Data Availability

The data presented in this study are available on request from the corresponding author.

## Supplementary Materials

The following are available online at https://www.mdpi.com/article/10.3390/polym13071032/s1, Figure S1: ^1^H-NMR spectrum of 60EG_4_; Figure S2: ^1^H-NMR spectrum of 60EG_4_-400BMA; Figure S3: (a) thermo-responsive polymer degree of polymerization calculated via ^1^H-NMR (*n*_NMR_) as a function of the EG_4_/CTA mole ratio (*n*_target_). (b) hydrophobic block degree of polymerization calculated via ^1^H-NMR (pNMR) as function of the BMA/macro CTA mole ratio (ptarget); Figure S4: Phase diagram reporting the reversibility of the phase separation as a function of the NP concentration and p in the case of *n* equal to 40 (a), 60 (b), 80 (c) and 100 (d), being RP = Reversible Precipitate, IP = Irreversible Precipitate, IG = Irreversible Gel and RG = Reversible Gel, respectively; Figure S5: NP forming a precipitate (left) or a hydrogel (right) once the temperature is increased above the Tcp; Figure S6: NP size as function of temperature during heating (triangle) and cooling (star) in the case of 60EG_4_-150BMA; Figure S7: NP size as function of block copolymers HLB in the case of *n* equal to 40 (square), 60 (circle), 80 (triangle) and 100 (star); Table S1: conversion (X), degree of polymerization (*n*), number-average molecular weight (Mn) and dispersity (Đ) of the macro CTAs synthesized; Table S2: conversion (X), degree of polymerization (*n*, *p*), number-average molecular weight (Mn) and dispersity (Đ) of the block copolymers synthesized; Equation (S1): EG_4_ conversion; Equation (S2): Degree of polymerization for the macro CTA from the corresponding ^1^H-NMR spectrum; Equation (S3): BMA conversion; Equation (S4): Degree of polymerization for the polyBMA from the corresponding ^1^H-NMR spectrum.
