# Supplementary material for: Influence of the Polymer Microstructure over the Phase Separation of Thermo-Responsive Nanoparticles
Source: Polymers (Basel). 2021 Mar 26;13(7):1032. doi: 10.3390/polym13071032 (PMC8037153; doi:10.3390/polym13071032)
Supplement: Supplementary file 1 [file polymers-13-01032-s001.pdf]

Supporting information for

Influence of the Polymer Microstructure over the Phase Separation of Thermo-Responsive Nanoparticles

Nicolò Manfredini, Marco Tomasoni, Mattia Sponchioni and Davide Moscatelli*

Department of Chemistry, Materials and Chemical Engineering "Giulio Natta", Politecnico di Milano, via Mancinelli 7, 2013 Milano – Italy.

*Correspondence to: Mattia Sponchioni; e-mail: mattia.sponchioni@polimi.it

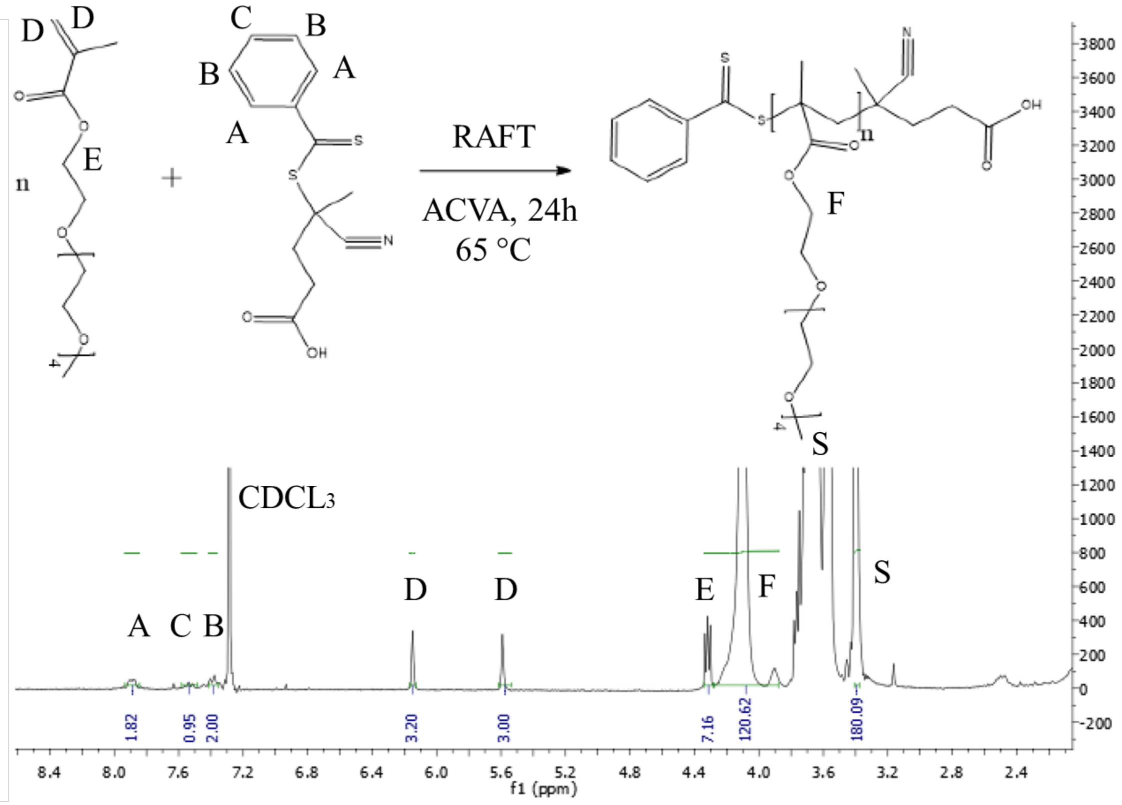

Figure S1: *H-NMR spectrum of 60EG4.*

The monomer conversion was calculated from the ^{1}H NMR spectra following **eq. S1**:

$$X = \frac{F}{E + F} \tag{S1}$$

Where X is the monomer conversion, E is the area of the peak attributed to the two hydrogens close to the oxygen in the unreacted monomer, F is the area of the peak associated to the hydrogens near the oxygen in the polymer (see labeling in **Figure S1**).

The degree of polymerization (*n*) of the EG_4MA was calculated according to **eq. S2:**

$$n = \frac{F}{B} \tag{S2}$$

With B being the two hydrogens associated to the aromatic ring in the RAFT agent.

Table S1: *conversion (X), degree of polymerization (n), number-average molecular weight (Mn) and dispersity (Đ) of the macro CTAs synthesized.*

Sample	**X** [%]	***n*** [-]	***Mn*** [Da]	***Đ*** [-]
**$40EG_4$**	98.2	41	12089	1.07
**$60EG_4$**	94.4	60	17268	1.22
**$80EG_4$**	98.7	85	28197	1.07
**$100EG_4$**	98.5	112	33467	1.08

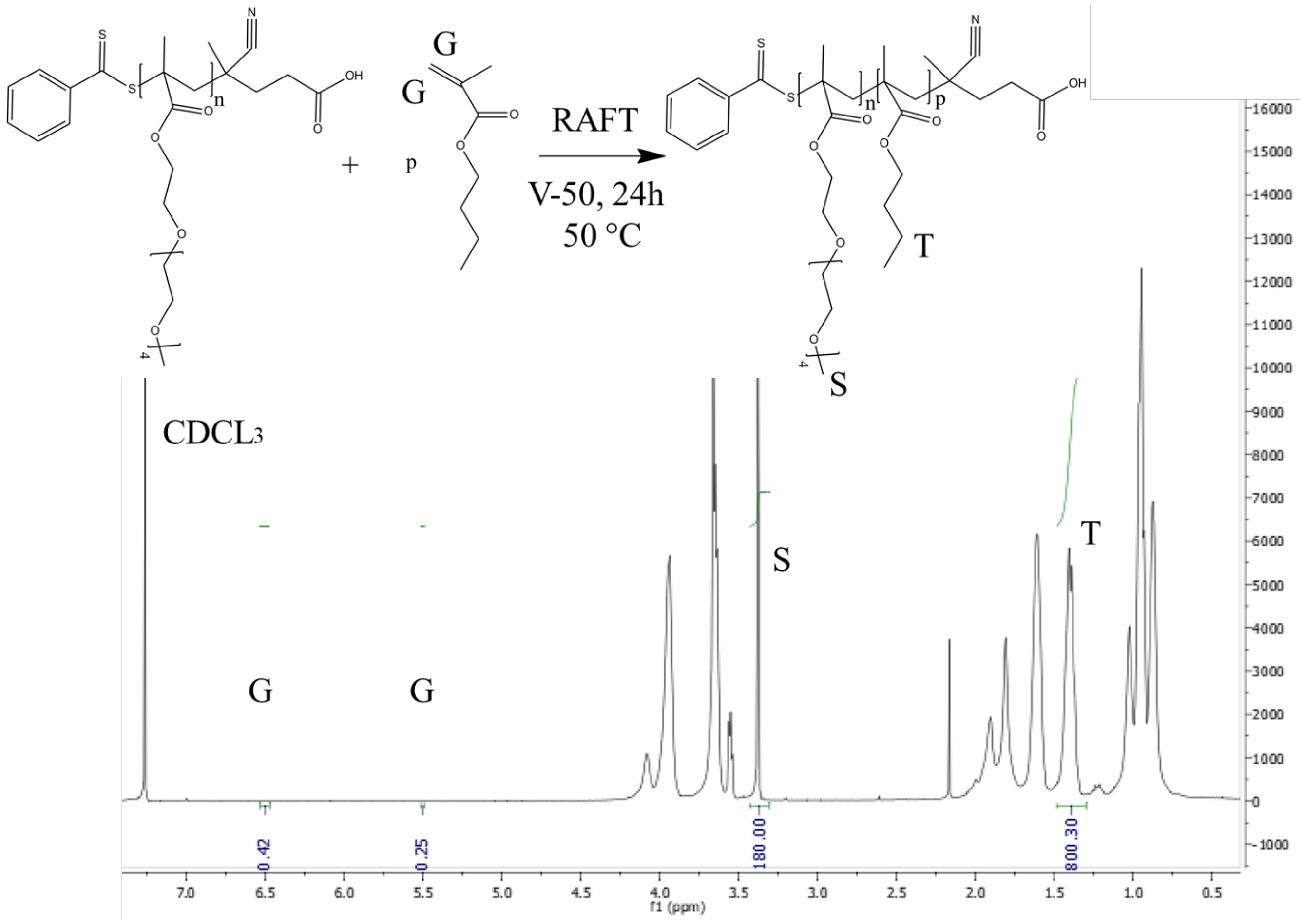

Figure S2: *H-NMR spectrum of 60EG$_4$-400BMA.*

The monomer conversion was calculated according to **eq. S3**:

$$X = \left(1 - \frac{2G}{T}\right) * 100 \quad (S3)$$

Where X is the monomer conversion, T is the area of the peak associated with the hydrogens near the methyl group either in the monomer or in the polymer and G is the area of the peak attributed to the two vinyl hydrogens in the unreacted monomer (see labeling in **Figure S2**).

The degree of polymerization (*p*) of the BMA was calculated according to **eq. S4:**

$$p = \frac{\frac{T}{2}}{\frac{S}{3n}} = \frac{3nT}{2S} \tag{S4}$$

Table S2: *conversion (X), degree of polymerization (n, p), number-average molecular weight (Mn) and dispersity (Đ) of the block copolymers synthesized.*

Sample	**X** [%]	***n*** [-]	***p*** [-]	***Mn*** [Da]	***Đ*** [-]
**40EG4-100BMA**	99.3	41	103	27464	1.18
**40EG4-200BMA**	98.8	41	208	47094	1.02
**40EG4-300BMA**	98.5	41	297	50569	1.38
**40EG4-500BMA**	98.7	41	520	103579	1.35
**40EG4-1000BMA**	99.1	41	980	194459	1.28
**40EG4-3000BMA**	98.0	41	2970	478350	1.19
**60EG4-150BMA**	99.9	60	145	41869	1.06
**60EG4-275BMA**	95.5	60	280	69969	1.14
**60EG4-400BMA**	97.4	60	400	82123	1.08
**60EG4-725BMA**	96.0	60	730	157002	1.10

**80EG4-450BMA**	95.0	85	425	108473	1.19
**80EG4-650BMA**	99.0	85	671	174748	1.19
**80EG4-850BMA**	99.0	85	848	228824	1.08
**100EG4-275BMA**	98.0	112	272	73899	1.38
**100EG4-600BMA**	93.3	112	620	168251	1.16
**100EG4-850BMA**	99.0	112	871	242335	1.14
**100EG4-1200BMA**	90.0	112	1190	312335	1.19

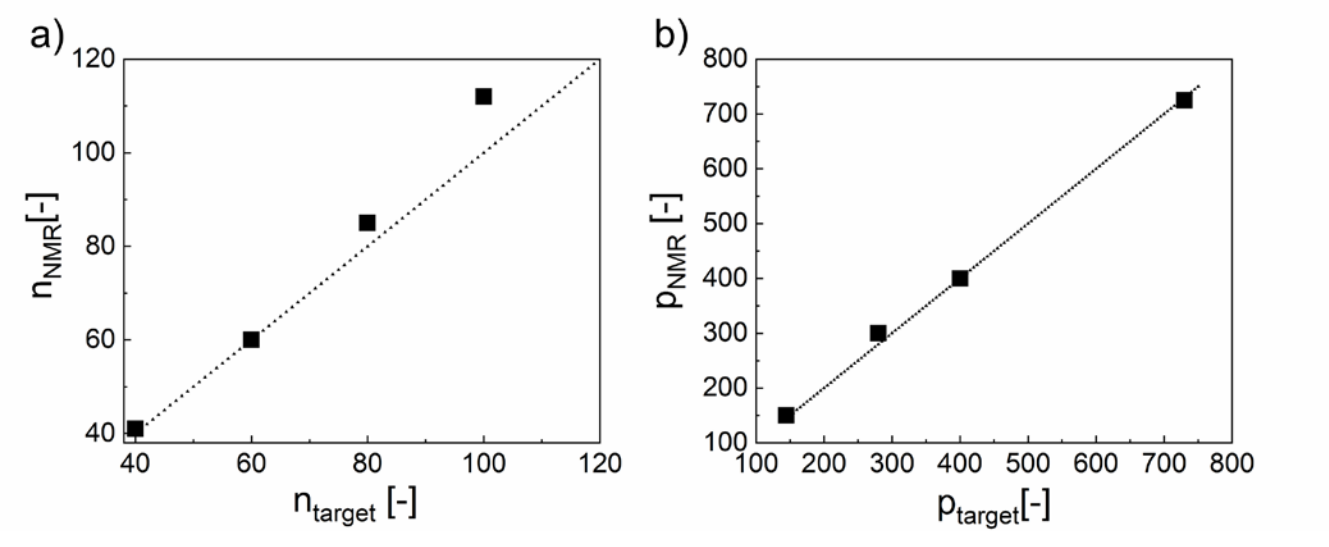

Figure S3: *(a) thermo-responsive polymer degree of polymerization calculated via* ^{1}*H-NMR (*n_{NMR}*) as a function of the EG4/CTA mole ratio (*n_{target}*). (b) hydrophobic block degree of polymerization calculated via* ^{1}*H -NMR (*p_{NMR}*) as function of the BMA/macro CTA mole ratio (*p_{target}*).*

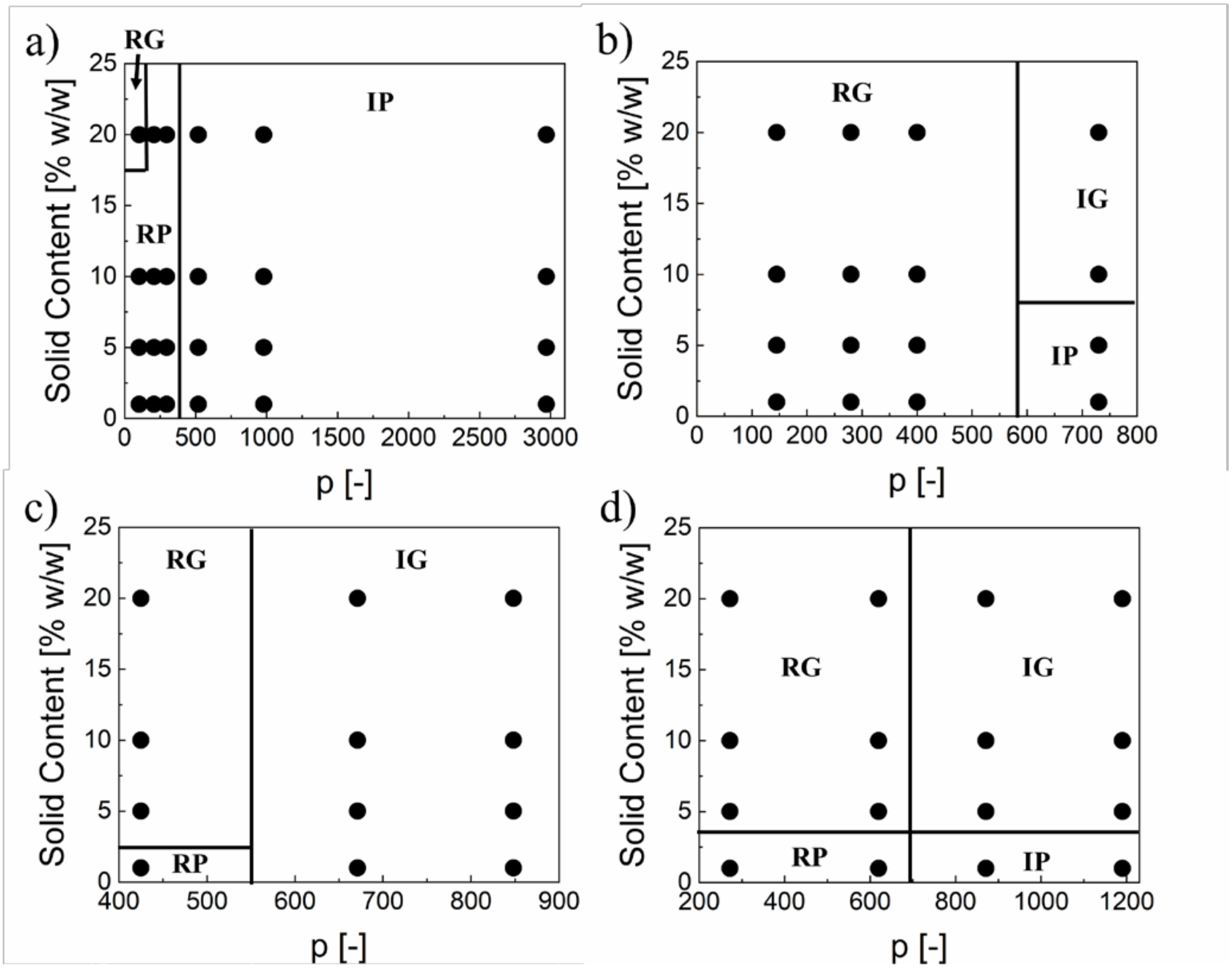

Figure S4: *Phase diagram reporting the reversibility of the phase separation as a function of the NP concentration and p in the case of n equal to 40 (a), 60 (b), 80 (c) and 100 (d), being RP=Reversible Precipitate, IP=Irreversible Precipitate, IG= Irreversible Gel and RG=Reversible Gel, respectively.*

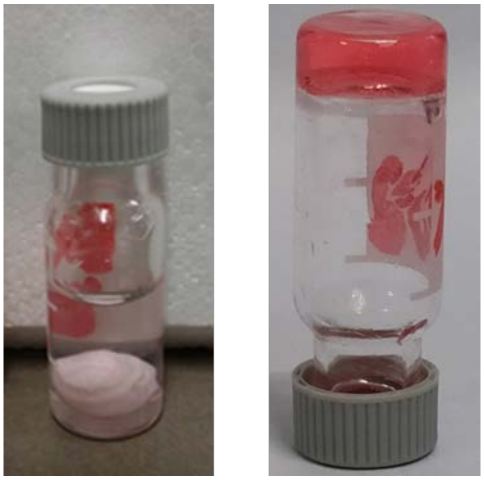

Figure S5: *NP forming a precipitate (left) or a hydrogel (right) once the temperature is increased above the Tcp.*

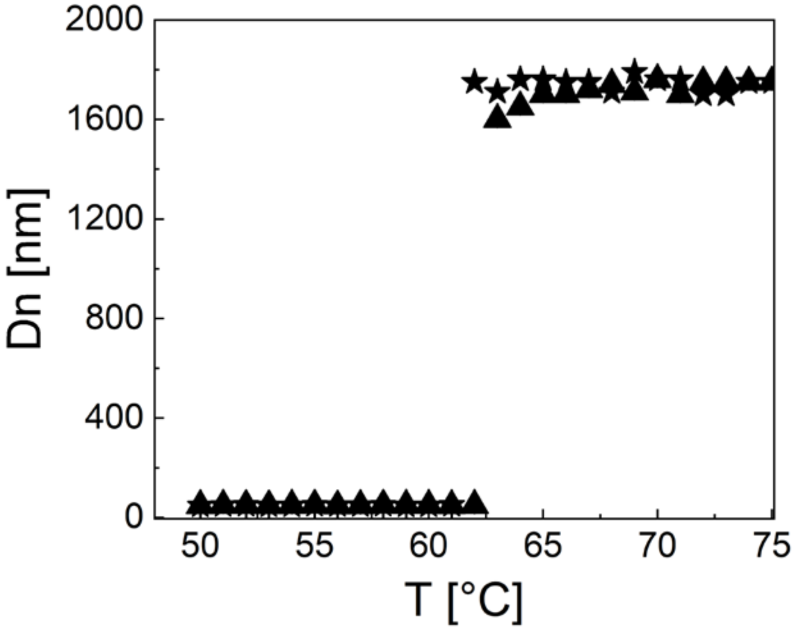

Figure S6: *NP size as function of temperature during heating (triangle) and cooling (star) in the case of 60EG4-150BMA.*

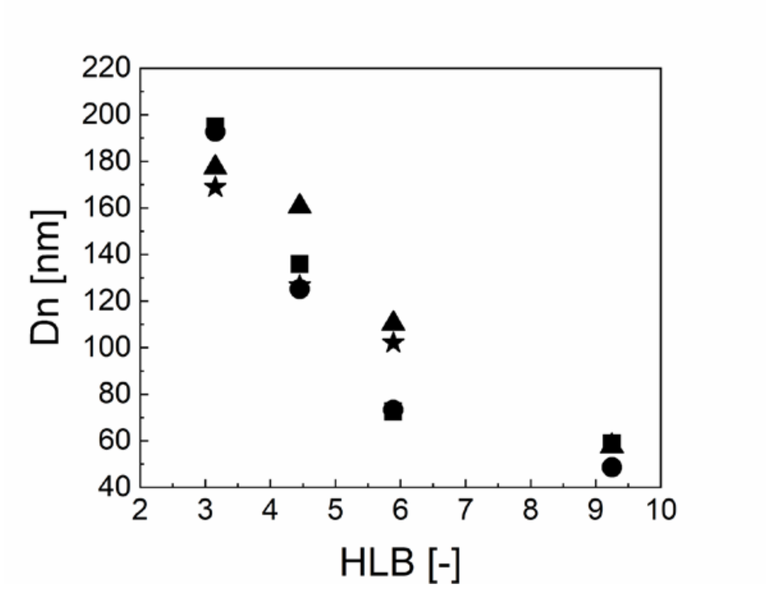

Figure S7: *NP size as function of block copolymers HLB in the case of n equal to 40 (square), 60 (circle), 80 (triangle) and 100 (star).*
